# Autophagic Cell Death by *Poncirus trifoliata* Rafin., a Traditional Oriental Medicine, in Human Oral Cancer HSC-4 Cells

**DOI:** 10.1155/2015/394263

**Published:** 2015-06-29

**Authors:** Hye-Yeon Han, Bong-Soo Park, Guem San Lee, Seung-Hwa Jeong, Hyungwoo Kim, Mi Heon Ryu

**Affiliations:** ^1^Department of Oral Pathology, School of Dentistry, Institute of Translational Dental Sciences, Pusan National University, Yangsan, Republic of Korea; ^2^Department of Oral Anatomy, School of Dentistry, Pusan National University, Yangsan, Republic of Korea; ^3^Department of Herbology, College of Korean Medicine, Wonkwang University, Iksan, Republic of Korea; ^4^Department of Preventive and Community Dentistry, School of Dentistry, Pusan National University, Yangsan, Republic of Korea; ^5^Division of Pharmacology, School of Korean Medicine, Pusan National University, Yangsan, Republic of Korea

## Abstract

*Poncirus trifoliata* Rafin. has long been used as anti-inflammatory and antiallergic agent to treat gastrointestinal disorders and pulmonary diseases such as indigestion, constipation, chest fullness, chest pain, bronchitis, and sputum in Korea. *P. trifoliata* extract has recently been reported to possess anticancer properties; however, its mechanisms of action remain unclear. In this study, its antiproliferative effects and possible mechanisms were investigated in HSC-4 cells. The methanol extract of *P. trifoliata* (MEPT) significantly decreased the proliferation of HSC-4 cells (inhibitory concentration (IC)_50_ = 142.7 *μ*g/mL) in a dose-dependent manner. While there were no significant changes observed upon cell cycle analysis and ANNEXIN V and 7-AAD double staining in the MEPT-treated groups, the intensity of acidic vesicular organelle (AVO) staining and microtubule-associated protein 1 light chain (LC) 3-II protein expression increased in response to MEPT treatment. Furthermore, 3-methyladenine (3-MA, autophagy inhibitor) effectively blocked the MEPT-induced cytotoxicity of HSC-4 cells and triggered the activation of p38 and extracellular signal-regulated kinases (ERK) proteins. Taken together, our results indicate that MEPT is a potent autophagy agonist in oral cancer cells with antitumor therapeutic potential that acts through the mitogen-activated protein kinase (MAPK) pathway.

## 1. Introduction

The immature fruit of* Poncirus trifoliata* Rafin. has long been used as a medicinal herb in Korea for treatment of gastrointestinal disorders, inflammation, allergies, and pulmonary diseases such as chest fullness, chest pain, bronchitis, and sputum [1–3]. In the theory of traditional medicine, the immature fruit of* P. trifoliata* can break stagnation of qi and remove food retention, resolve phlegm, and eliminate mass [2]. Accordingly, it is used to treat indigestion, constipation due to accumulation of heat, and dysentery [2].

*P. trifoliata* was recently reported to have various properties, such as antibacterial, antiallergic, and anti-tumor activities [1, 4, 5], and it is known to contain limonin, imperatorin, 25-Methoxyhispidol A, beta-sitosterol, 2-hydroxy-1,2,3-propanetricarboxylic acid 2-methyl este, neohesperidin, and poncirin [6–9]. However, the antitumor effects of* P. trifoliata* extracts on oral cancer and the molecular mechanisms underlying their antitumor activities are not fully understood.

Oral squamous cell carcinoma (OSCC) is the sixth most common cancer in the world [10, 11]. The treatment modalities for OSCC are usually a combination of surgery, chemotherapy, and radiation to decrease the possibility of distant metastasis. Despite the combined therapies for OSCC, the 5-year survival rate is only around 50% [12], and OSCC patients suffer from posttherapeutic complications, including facial deformities, osteonecrosis, and life-threatening side effects of the chemotherapeutic regimen [13]. Therefore, the discovery and development of alternative therapeutic strategies for the treatment of OSCC is highly desirable.

Autophagy is an evolutionarily conserved catabolic pathway involved in lysosomal degradation of long-lived microorganelles and turnover of cellular proteins and macromolecules; therefore, it is regarded as a survival and protective mechanism [14]. However, excessive and sustained autophagy can modulate nonapoptotic programmed cell death [15]. In addition, the role of autophagy in cancer cells remains controversial, and there is debate regarding whether it protects cancer cells from apoptosis or induces cell death under genotoxic stress. Recent studies have demonstrated that chemotherapeutic stress can trigger autophagic cell death in various cancer cells, which can be an alternative to current cancer therapies, especially in cases of apoptosis-resistant cancer cells [16].

The present study further evaluated the antitumor effects of methanol extract of* P. trifoliata* (MEPT) on OSCC cells. The results revealed the role of autophagy induced by MEPT and reviewed the findings of oriental herbal medicine and autophagy. In addition, we explored the molecular mechanisms of MEPT-induced autophagy in HSC-4 cells.

## 2. Materials and Methods

### 2.1. Reagents and Antibodies

Paclitaxel, trifluoperazine (TFP, an activator of autophagy), MTT (3,4,5-dimethyl N-methylthiazol-2-yl-2, 5-d-phenyl tetrazolium bromide), propidium iodide (PI) solution, acridine orange (employed for acidic vesicular organelle (AVO) staining), primary antibody against microtubule-associated protein 1 light chain (MAP1-LC; also known as LC) 3, cell culture medium supplements (insulin, apo-transferrin, triiodothyronine, hydrocortisone, and cholera toxin) and 3-methyladenine (3-MA) were purchased from Sigma-Aldrich (St. Louis, MO, USA). Anti-mouse IgG antibody and anti-rabbit IgG antibody were obtained from Enzo Life Sciences (Farmingdale, NY, USA). Primary antibodies for c-Jun *N*-terminal kinases (JNK), phospho-JNK, p38, and phospho-p38 were obtained from Cell Signaling Technology (Beverly, MA, USA), while beta-actin, ERK, and phospho-ERK were purchased from Santa Cruz Biotechnology (Santa Cruz, CA, USA). In addition, antibodies for autophagy-related gene and proteins (Atg) 4B and Atg5- Atg12 were acquired from Abcam (Cambridge, UK).

### 2.2. Preparation of MEPT

The immature fruit of* P. trifoliata* (dried fruit) was purchased from Hwalim Medicinal Herbs (Pusan, Korea). Extraction was conducted using our standard procedure [17]. Briefly, 50 grams of crude drug was immersed in one liter of methanol, sonicated for 30 min, and then extracted for 48 h. The obtained extract was then filtered using number 20 Whatman filter paper, evaporated under reduced pressure using a vacuum evaporator (Eyela, Tokyo, Japan), and lyophilized using a freeze dryer (Labconco, Kansas City, MO, USA). Finally, 8.66 g of lyophilized powder was obtained (yield, 17.32%). A sample of the lyophilized powder (MEPT, Voucher number MH2013-007) and specimen (Voucher number MS2013-007) was deposited at the Division of Pharmacology, School of Korean Medicine, Pusan National University (see Supplementary Material available online at http://dx.doi.org/10.1155/2015/394263).

### 2.3. Cell Culture and Treatment of MEPT

HSC-4 cells (human oral squamous cell carcinoma cell line) were maintained in culture medium composed of Dulbecco Modified Eagle Medium (DMEM) and Ham's F-12 media (at a ratio of 3 : 1) supplemented with 10% fetal bovine serum (FBS), insulin, apo-transferrin, triiodothyronine, hydrocortisone, cholera toxin, and 1% penicillin/streptomycin at 37°C in an incubator with 5% CO_2_ humidified atmosphere. Equal numbers of cells (5 × 10^4^ cells/well) were seeded in 24-well plates and allowed to attach, after which cells were treated with MEPT at 0, 25, 50, 100, or 200 *μ*g/mL. Untreated cells of control group were treated with the same volume of absolute ethanol (vehicle). After 24 h of treatment, cells were submitted for MTT assays and other analyses.

### 2.4. Determination of Cell Viability with MTT Assay

Cell viability in response to MEPT treatment was evaluated using an MTT assay. Briefly, MTT solution was diluted in DMEM (1 : 9), after which 500 *μ*L were added to each well and the cells were incubated at 37°C for 4 h in a 5% CO_2_ atmosphere. The absorbance of converted dye was then measured at 570 nm using a microplate reader (Bio-Rad Laboratories, Hercules, CA, USA).

### 2.5. Observation of Cell Morphology

The morphologies of untreated and MEPT-treated HSC-4 cells were observed with a phase contrast microscope (Olympus, Tokyo, Japan). Photographs were taken and the morphological changes were analyzed.

### 2.6. Cell Cycle Analysis

Cell cycle distributions were evaluated by flow cytometry to analyze the extent of apoptosis and necrosis and identify the type of cell death. Briefly, cells were seeded in 24-well plates (5 × 10^4^ cells per well) and incubated with MEPT at 0, 25, 50, or 100 *μ*g/mL for 24 h. Following MEPT treatment, cells were trypsinised, fixed, and stained with PI solution (10 *μ*g/mL) at 4°C for 30 min. Fluorescence intensities were measured using a FACScan flow cytometer (BD Bioscience, Heidelberg, Germany).

### 2.7. ANNEXIN V and 7-AAD Double Staining

Cells were seeded in 6-well plates (3 × 10^5^ per well), incubated overnight and treated with MEPT at the indicated concentrations for 24 h. The cells of control group were treated with vehicle for 4 h. Paclitaxel (30 nM) was used as a positive control. After incubation, apoptotic cells were detected using an ANNEXIN V-FITC apoptosis detection kit (Enzo Life Sciences) according to the manufacturer's instructions. Stained cells were analyzed using a flow cytometer (BD Biosciences, Heidelberg, Germany) and the data obtained was analyzed using the FACSCanto II (Fluorescence Activated Cell Sorting) software.

### 2.8. AVO Staining

AVO staining was conducted to detect the presence of acidic vesicles after MEPT treatment. Briefly, cells were treated with a final concentration of 1 *μ*M of acridine orange solution at 37°C for 15 min, washed in phosphate buffered saline (PBS), and observed under a fluorescence microscope (excitation = 488 nm; emission = 520 nm) (Carl Zeiss, Germany).

### 2.9. Western Blot Analysis

After MEPT treatment, cell lysates were prepared with RIPA buffer (Cell Signaling Technology) according to the manufacturer's instructions. The same amount of protein (50 *μ*g) from each sample was separated in SDS-polyacrylamide gels. After being transferred to polyvinylidene fluoride membranes and blocking with skim milk, the membranes were incubated at 4°C overnight with primary antibodies against JNK, phospho-JNK, p38, phospho-p38, ERK, phospho-ERK, and beta-actin (the internal control). The HRP-conjugated secondary antibody (1 : 8000 dilution) was applied at room temperature. Bound antibodies were detected using SuperSignal West-Femto reagent (Pierce, Rockford, IL, USA).

### 2.10. Statistical Analysis

Data were analyzed by Student's *t*-test using Window PASW (Predictive Analytics SoftWare) version 21.0 (SPSS Inc., Armonk, NY, USA) to identify significant differences between the control and experimental groups. The data were expressed as the means ± standard deviation (SD), and a *P* < 0.05 was considered to be statistically significant.

## 3. Results

### 3.1. Effects of MEPT on Proliferation Rates and Morphologic Changes

**Figure 1:**
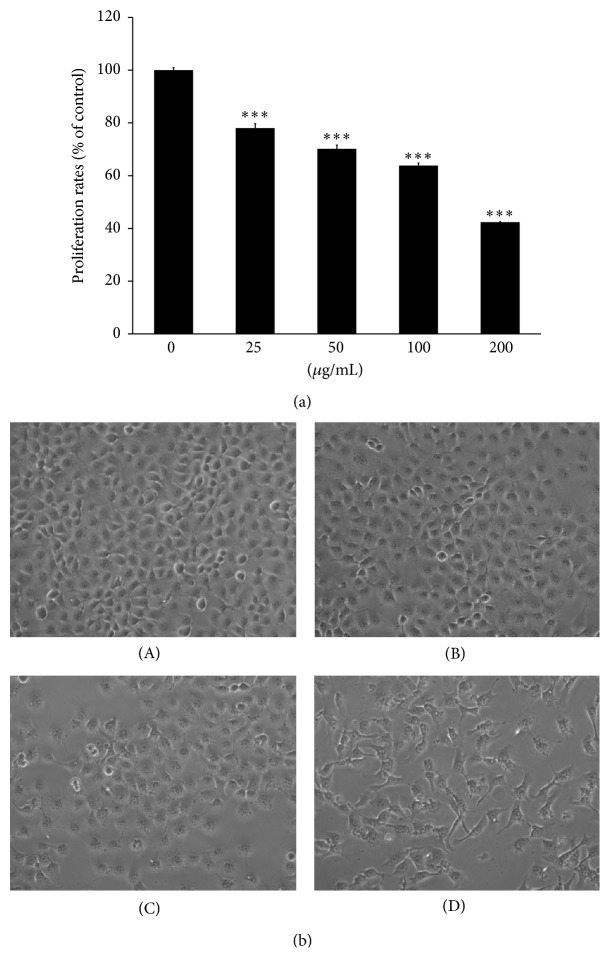
MEPT-induced inhibition of proliferation and changes in cell morphology. HSC-4 cells treated with 0, 25, 50, 100, or 200 *μ*g/mL of MEPT for 24 h were analyzed using an MTT assay (a). MEPT caused a change in cell morphology consistent with autophagy in HSC-4 cells (original magnification, ×200). Cells were treated with (A) 0 *μ*g/mL, (B) 25 *μ*g/mL, (C) 50 *μ*g/mL, or (D) 100 *μ*g/mL of MEPT for 24 h (b). Results are presented as the means ± standards deviation of three independent experiments. ^*∗∗∗*^ *P* < 0.001 versus nontreated control.

MEPT treatment for 24 hours restricted the proliferation rates of HSC-4 cells in a dose-dependent manner (Figure 1(a)) with inhibitory concentration (IC)_50_ values of 142.7 *μ*g/mL at 24 h. After treatment, numerous HSC-4 cells had shrunk, become flattened, and developed intracytoplasmic vacuoles. In addition, a decrease in cell density was observed in the 50 and 100 *μ*g/mL MEPT-treated groups (Figure 1(b)).

### 3.2. Effects of MEPT on Cell Cycle Distribution

**Figure 2:**
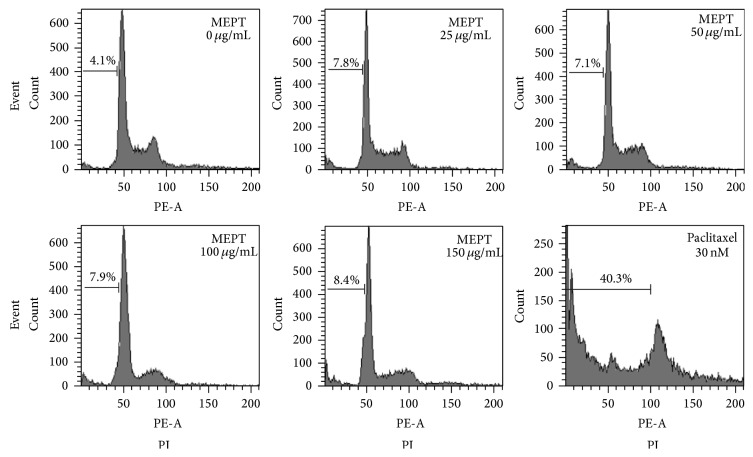
Cell cycle analysis of MEPT-treated HSC-4 cells. Cells were treated with various concentrations of MEPT for 24 h or with 30 nM of paclitaxel for 24 h, after which cell cycle distributions were analyzed by flow cytometry.

In the normal group, 4.1% of cells were in the sub-G1 phase. Cells treated with MEPT for 24 h showed no significant increase in sub-G1 arrest. The proportions of cells in sub-G1 arrest in the 25, 50, 100, or 150 *μ*g/mL of MEPT-treated group were almost the same (Figure 2).

### 3.3. Evaluation of Early and Late Apoptosis in MEPT-Treated HSC-4 Cells

**Figure 3:**
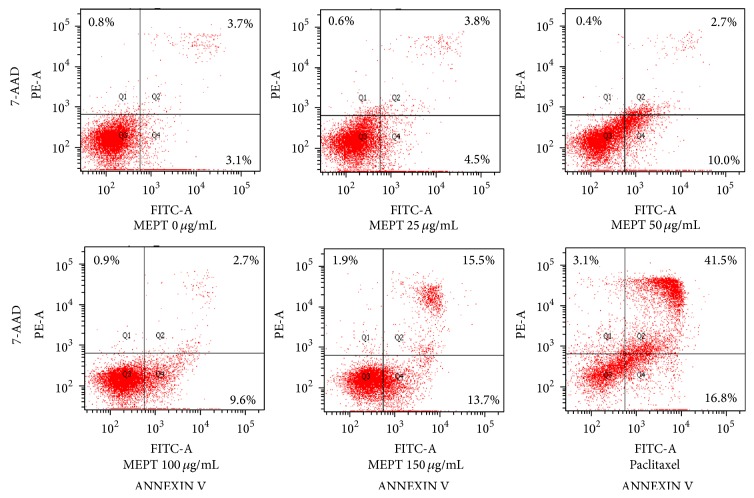
Effects of MEPT on the induction of apoptosis. HSC-4 cells were treated with 0, 25, 50, 100, or 150 *μ*g/mL of MEPT for 24 h or 30 nM paclitaxel for 24 h. Cells were stained with ANNEXIN V and 7-AAD and analyzed by flow cytometry. Early apoptotic cells were stained by ANNEXIN V, but not by 7-AAD (lower right quadrant, Q4), whereas late apoptotic cells were stained by both of ANNEXIN V and 7-AAD (upper right quadrant, Q2).

The number of apoptotic cells in the MEPT-treated groups did not increase significantly. HSC-4 cells treated with 150 *μ*g/mL of MEPT showed early and late apoptosis proportions of 13.7% and 15.5%, respectively, whereas treatment with paclitaxel at 30 nM caused apoptosis of 16.8% and 41.5% of cells, respectively (Figure 3).

### 3.4. Induction of Autophagy in MEPT-Treated HSC-4 Cells as Determined by AVO Staining

**Figure 4:**
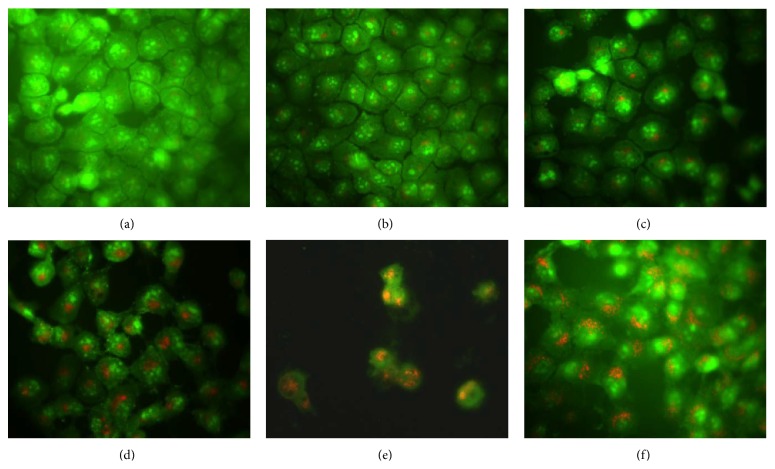
Effects of MEPT on the formations of acidic vesicular organelle (AVO) in HSC-4 cells. Cells were treated with vehicle or 25, 50, 100, or 150 *μ*g/mL of MEPT for 24 h, then stained with acridine orange, and visualized by fluorescent microscopy. AVO staining at MEPT concentrations of (a) 0 *μ*g/mL; (b) 25 *μ*g/mL; (c) 50 *μ*g/mL; (d) 100 *μ*g/mL; (e) 150 *μ*g/mL; and (f) Earle's balanced salt solution (EBSS, positive control for autophagy) (original magnification, ×400).

As shown in Figure 4, AVO formation was not detected in the vehicle-treated group or 25 *μ*g/mL MEPT-treated cells. However, distinct punctate formation, the hallmark of autophagy induction, was observed in HSC-4 cells treated with >50 *μ*g/mL MEPT (Figure 4).

### 3.5. Activation of LC3 and Atg Proteins following MEPT Treatment

**Figure 5:**
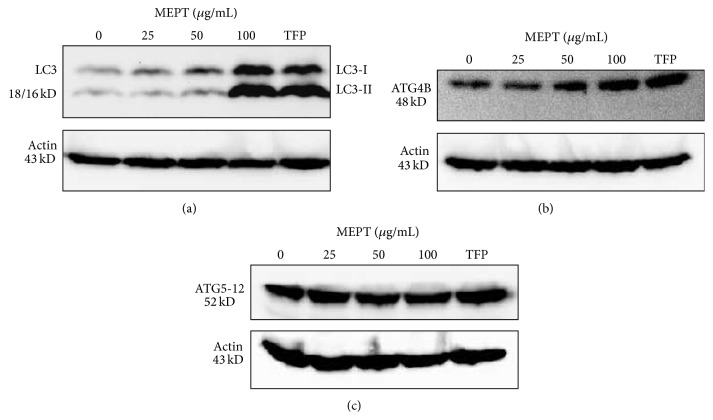
The expression of LC3 and Atg proteins in MEPT-treated HSC-4 cells. Western blotting was performed for LC3-I and LC3-II, Atg4B, and Atg5-12 conjugate protein after administering MEPT at 0, 25, 50, or 100 *μ*g/mL for 24 h or 10 *μ*M TFP (positive control for autophagy) for 24 h.

As shown in Figure 5, MEPT treatment converted LC3-I into LC3-II isoform and activated Atg4B protein. However, the level of Atg5-12 conjugate was not affected by MEPT (Figure 5).

### 3.6. Alterations in MEPT-Treated Cell Viability following 3-MA Pretreatment

**Figure 6:**
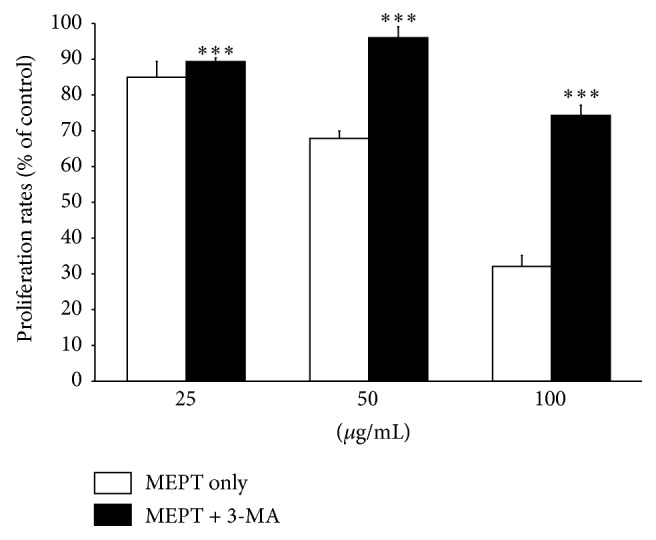
Effects of MEPT on the viabilities of HSC-4 cells pretreated with 3-MA. HSC-4 cells were pretreated with or without 3-MA for 1 h and then 25, 50, or 100 *μ*g/mL of MEPT for 24 h, after which cell viability was analyzed using an MTT assay. Results are expressed as the means ± standard deviations. ^*∗∗∗*^ *P* < 0.001 versus the same amount of MEPT-treated cells.

To determine the type of MEPT-induced autophagy, we conducted an MTT assay of HSC-4 cells pretreated with 3-MA (a classical inhibitor of autophagy) for 1 h prior to MEPT treatment. The results revealed that the cell viability of the 3-MA pretreated and MEPT-treated cells was significantly greater than that of only MEPT-treated cells (Figure 6).

### 3.7. Triggering of the p38 and ERK Pathway by MEPT Treatment

**Figure 7:**
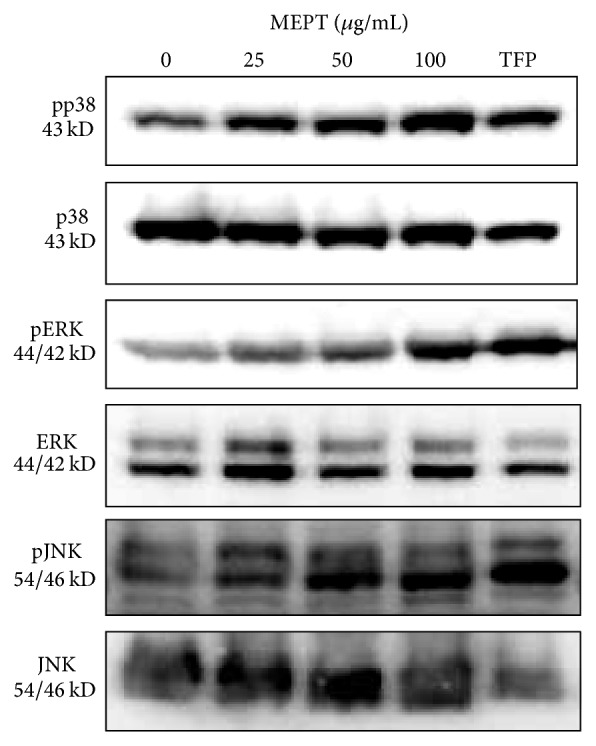
Expression and activation of MAPK proteins in MEPT-treated HSC-4 cells. Western blot analysis was conducted to measure the total and phosphorylated forms of p38, ERK, and JNK in HSC-4 cells treated with 0, 25, 50, or 100 *μ*g/mL of MEPT for 24 h or 10 *μ*M TFP for 24 h. The expression levels of phospho-p38, phospho-ERK, and phospho-JNK were normalized using a loading control.

MEPT induced the phosphorylation of p38 and ERK in a dose-dependent manner but had no effect on phospho-JNK levels (Figure 7).

## 4. Discussion

The objective of this study was to investigate the antitumor effects of MEPT on human OSCC cells and explore the mechanism by which it induces cell death. Previous studies demonstrated that MEPT exerted a remarkable dose-dependent inhibitory effect on the proliferation of many cancer cell lines [1, 3, 7, 8]. The results of the present study showed that MEPT can potentially suppress the proliferation of HSC-4 cells, eventually leading to cell death, which was identified as autophagic cell death through positive AVO staining, an increased LC3-I/LC3-II ratio, Atg4B protein, and recovered viability in inhibitor study using 3-MA. In addition, MEPT also activated p38 and ERK pathways. These results suggest the possible use of MEPT for the treatment of OSCC.

Autophagy can be regulated by a number of Atgs, including Atg4, Atg5, Atg8, and Atg12 [18]. Atg4 and Atg5 are involved in the elongation of autophagosomal vesicles [19]. More importantly, cysteine protease Atg4 catalyzes the proteolytic cleavage of pro-LC3 to generate LC3-I isoform, eventually forming membrane-bound LC3-II via the Atg5-Atg12-Atg16L complex [20–22]. The knockdown of Atg4 can accumulate LC3-II protein in cytosol and suppress autophagy because it plays a critical role in formation of LC3-I and recycling of LC3-II [19]. Moreover, previous reports indicated that Atg4B can be a potential biomarker and considered a more readily usable target of cancer therapy in autophagic machinery [19, 22]. Our results showed that the expression of the Atg4B protein increased in response to MEPT treatment, which triggered the induction of autophagic cell death in HSC-4 cells. Therefore, the modulation of Atg4B protein expression and activity can be an alternative strategy to traditional therapies for OSCC.

The MAPK pathway is a cascade of serine/threonine protein kinases that transmits a signal into cytosol from outside [23]. To further investigate the mechanism by which MEPT induces autophagic cell death, we conducted western blot analysis of p38 and ERK proteins. MAPK pathways have been reported to be implicated in the steps of autophagy. Choi et al. demonstrated that the p38 pathway controlled autophagy at the step of sequestration, while the ERK pathway controlled autophagy at the step of maturation [24]. Our results clarified that MEPT induces significant autophagic cell death via phosphorylation of p38 protein without the induction of apoptosis, indicating the therapeutic potential of MEPT in apoptosis-resistant cancers.

The result of the present study indicated that ERK protein was activated by MEPT treatment. Activation of the ERK pathway is believed to be implicated in cell proliferation and differentiation and the promotion of survival processes [23]. However, recent findings suggest that the role of the ERK pathway in autophagy regulation is controversial. Wang and Wu reported that cisplatin-induced autophagy is regulated by the ERK pathway [25]. In addition, Liu et al. and Singh et al. showed that activation of the ERK cascade promoted autophagic cell death and apoptosis, respectively [23, 26]. Furthermore, during autophagy, the activated ERK pathway has been implemented in the activation of Atg4B. Together with these findings, MEPT treatment can induce autophagic cell death, which is followed by activation of the ERK pathway in a dose-dependent manner.

The function of autophagy in cancer is very complicated and has become the object of controversy. Autophagy plays a critical role in tumor initiation and development. However, whether autophagy can play a role as a promoter or suppressor in tumorigenesis appears to depend on the cancer type, stage of cancer development, and genetic context [16, 27, 28]. Furthermore, autophagy and apoptosis can play opposing roles in cancer cells or both apoptotic and autophagic cell death, and they can occur simultaneously, or sequentially, or synergistically [28]. Several proteins can regulate both apoptosis and autophagy, as well as the modulation of autophagy through apoptotic signalling pathways [29]. For these reasons, investigation of the molecular mechanisms of autophagy and manipulation of autophagy induction for cancer cell death can be a key factor in an effective anticancer strategy.

Traditional medicines have long been used to treat cancer in Korea and are important sources of antitumor drugs [30]. Currently, medicinal herbs are employed as part of complementary or alternative medicine [31]. Numerous screening studies have shown that a panel of herbal medicines induced apoptosis [15, 30, 32]. However, accumulating evidence has shown that herbal medicines can trigger multiple pathways of cell death, including autophagic cell death and necrotic cancer cell death [14, 30]. As mentioned above, autophagic cell death can function as an alternative process of cancer cell death, and some herbs induce necrotic cell death in the absence of morphologic characteristics of apoptosis or autophagy [30]. More studies are needed to elucidate the detailed mode of action of herb-induced cell death at the molecular and cellular levels, which will provide valuable information for development of new effective chemotherapeutic drugs.

## 5. Conclusions

The results of the present study indicate that MEPT treatment effectively induces autophagic cell death, but not apoptosis, through activation of the p38 and ERK pathways in human oral cancer HSC-4 cells. Thus, our findings suggest that MEPT has the potential for use as a therapeutic agent for the treatment of oral cancer.

## Supplementary Material

Chromatograms of poncirin, standard material of *Poncirus trifoliata*, and MEPT at UV wavelength of 280 nm were shown in the supplementary Materials.

## Abbreviations

Atg: Autophagy-related gene and proteins
AVO: Acidic vesicular organelle
ERK: Extracellular signal-regulated kinases
JNK: c-Jun *N*-terminal kinases
LC3: Microtubule-associated protein 1 light chain 3
MAPK: Mitogen-activated protein kinases
MEPT: Methanol extract of* P. trifoliata*
OSCC: Oral squamous cell carcinoma.
