# Supplementary material for: Autophagic Cell Death by Poncirus trifoliata Rafin., a Traditional Oriental Medicine, in Human Oral Cancer HSC-4 Cells
Source: Evid Based Complement Alternat Med. 2015 Jun 29;2015:394263. doi: 10.1155/2015/394263 (PMC4499625; doi:10.1155/2015/394263)
Supplement: Supplementary file 1 — Chromatograms of poncirin, standard material of Poncirus trifoliata, and MEPT at UV wavelength of 280 nm were shown in the supplementary Materials. [file 394263.f1.pdf]

Supplementary data

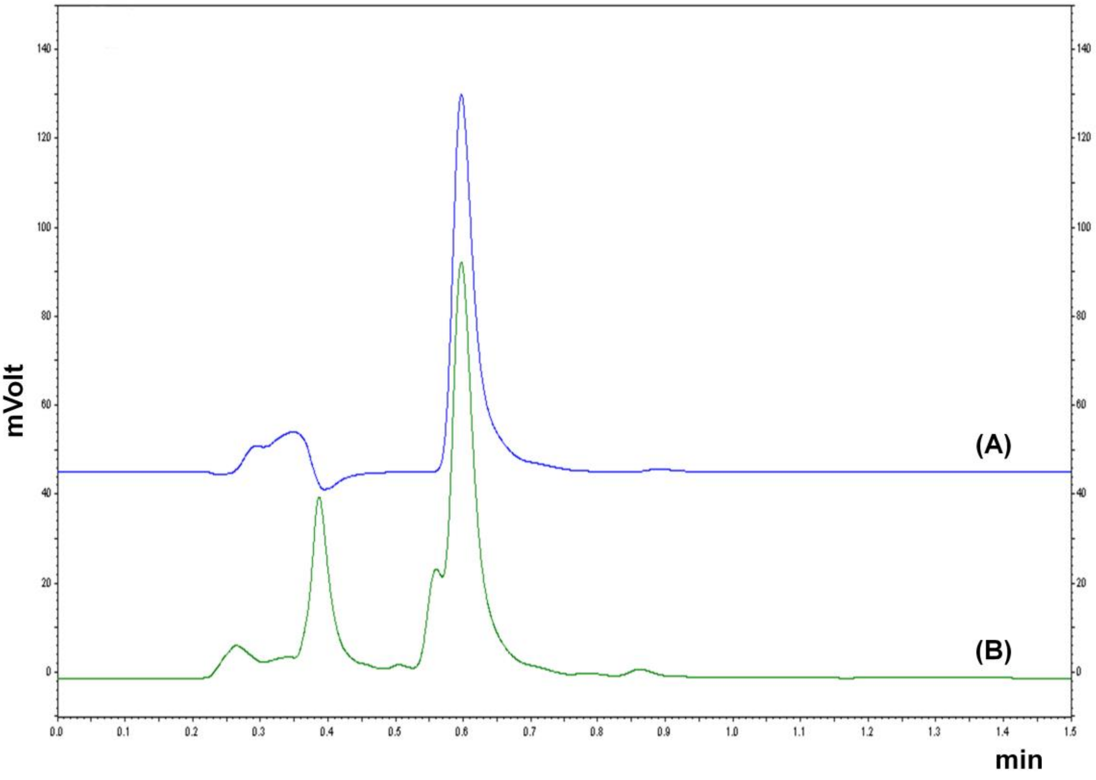

Fig. 1. Chromatograms of poncirin (A) and the methanol extract of *Poncirus trifoliata* (B) at UV wavelength of 280 nm.

Materials and Methods

Reagents

Methanol, acetonitrile and water were HPLC grade obtained from Fisher Scientific (Pittsburgh, PA, USA). Poncirin was purchased from Extrasynthese with a purity > 97.5% (GENAY cedex, France).

Chromatographic conditions and preparation of standard

Smart LC system comprised a LC800 (GL sciences, Japan) equipped with built-in apparatus including solvent delivery unit, autosampler, column oven and UV-visible detector. The acquired data was processed using EZChrom Elite software (Ver. 3.3.2 SP1). Chromatographic separation was performed on a Inertsil ODS-4 column (2.1 x 50 mm, 2 μm; GL sciences, Japan) with the temperature at 35 ℃. The mobile phase consisted of water (A) and acetonitrile (B). A gradient program of mobile phase was used as follows: 5% (B) maintained for 5 min, 5–90% (B) over 5–7 min. The flow rate

was set at 0.4 mL/min and the injection volume was 1 μL. Detection wavelength of poncirin was set at 280 nm. Poncirin was used as standard material of *Poncirus trifoliata*, one milligram of poncirin was accurately weighed and dissolved in methanol at the concentration of 100 μg/mL and the solution was 10-fold diluted before the injection.
